# Adult-Onset Still’s Disease: Typical Presentation, Delayed Diagnosis

**DOI:** 10.7759/cureus.8510

**Published:** 2020-06-08

**Authors:** Tiago Seco, Ana Cerqueira, Ana Costa, Carlos Fernandes, Jorge Cotter

**Affiliations:** 1 Internal Medicine, Hospital Senhora Da Oliveira, Guimarães, PRT

**Keywords:** adult-onset still’s disease, fever, rash, arthritis

## Abstract

Adult-onset Still’s disease (AOSD) is an uncommon auto-inflammatory disease of unknown etiology, with a classical triad of fever, arthritis, and evanescent rash. Its low prevalence and lack of specific guidelines contribute to frequent delays in diagnosis and treatment. Clinical manifestations vary greatly between mainly systemic or articular symptoms and the clinical pattern between monocyclic, polycyclic, or chronic illness. Treatment options include non-steroid anti-inflammatory drugs (NSAIDs), systemic corticoids, disease-modifying anti-rheumatic drugs (DMARDs), and, more recently, biological agents directed at identified immune pathological pathways like anti-interleukin-1 (IL-1) or anti-tumor necrosis factor-alpha (TNF-alpha).

We report a case of a 40-year-old male with persistent fever, polyarthralgia, sore throat, and rash for two weeks despite antibiotic treatment for suspected bacterial pharyngitis. During hospitalization and after extensive diagnostic workup, an AOSD diagnosis was made according to Yamaguchi’s criteria and successfully managed with systemic corticoids.

## Introduction

Adult-onset Still’s disease (AOSD) is a rare systemic inflammation condition with an estimated incidence of approximately 0.16 cases in 100,000 people, characterized by a classic clinical triad of daily fever spikes, arthritis, and typical salmon-colored evanescent rash [1]. It was first described in 1971 by Bywaters [2] and owes its name to a similar syndrome reported in children in the previous century by George Still [3].

It has a slightly higher incidence in females, with a ratio of 3:2. Although incidence reaches peaks between 16 and 25 and 35 and 45 years, case reports refer to the diagnosis as late as in patients in their 80s [1].

Due to its low frequency, most studies on AOSD come from isolated clinical reports or small series limiting the research and the validity of the findings.

AOSD’s etiology remains unknown. It is now being described as a polygenic auto-inflammatory syndrome due to the importance of innate immune pathways activation [4]. One of the most accepted theories hypothesizes a genetic predisposition upon which an environmental trigger (probably mostly viral infections) acts, much like in the case of reactive arthritis [5].

AOSD can be further subclassified according to chronological evolution (as a single event, intermittent events separated by periods of total remission, or chronic illness) or according to the predominant symptomology (systemic or articular). Each subclassification prompts different treatment options [6]. The chronic subtype tends to mainly manifest through articular symptoms and carries a high risk of articular destruction.

Numerous diagnostic criteria sets have been proposed; the most widely used being the ones proposed by Yamaguchi et al. due to higher sensitivity and consisting of both clinical and analytical parameters [7]. Fautrel et al. presented their set of criteria, including serum ferritin, a well-recognized serologic marker of disease activity [8]. Most of the existing sets of criteria underline that the diagnosis of AOSD can only be established after the exclusion of other conditions. However, there are no guidelines suggesting the extension of the differential diagnosis list or a diagnostic workup to perform.

Regarding this limitation of previously validated sets, Crispin et al. proposed a clinical scale based on positive symptoms and analytical markers allowing for the diagnosis of AOSD, in the context of fever of unknown origin, without further investigation and with high sensitivity [9].

## Case presentation

A 40-year-old male presented to the emergency department (ED) with complaints of arthralgia, evanescent rash, sore throat, and fever. Two weeks prior, he developed a sore throat, arthralgia in both wrists, daily fever spikes, anorexia, and adynamia. After reporting these symptoms to his attending physician, he was prescribed two consecutive antibiotic courses for presumptive bacterial pharyngitis. Persisting symptoms led to the ER visit. He reported an unremarkable personal and family clinal history and no chronic medication (recent or otherwise). He was a non-smoker, without regular alcohol consumption, and denied having unprotected sex, with the exception of his partner. He lived in an urban environment with access to public distribution of water and with no contact with animals. In the ED, he was afebrile (temperature of 37.8ºC) and discrete pharyngeal erythema was noted, as well as arthralgia of both wrists and some interphalangeal joints of both hands without arthritis. Hematological analysis showed elevated leucocyte count with neutrophilia (17.7x10^9/L with 81.9% PMN cells), elevated C-reactive protein (CRP; 200 mg/L; normal <2.9 mg/L), and erythrocyte sedimentation rate (77 mm; normal range < 10 mm), elevated hepatic cytolysis markers (2-3 times the upper limit of normal), and serum ferritin >2,000 ng/mL. The Thoracic CT scan and abdominal echography were unremarkable. The patient was thus admitted for a complementary study of a febrile syndrome.

During the hospitalization, the patient maintained daily fever spikes (with temperature varying between 38.5ºC and 39.2ºC) with a short duration and no identifiable pattern. An evanescent, maculopapular, salmon-colored rash either appearing on the trunk or the limbs was observed following the fever spikes, as well as arthritis of both wrists, most interphalangeal joints in both hands, and the left ankle. Further studies reported no microbiological isolates (including a search for acid alcohol-resistant bacilli) in either blood, urine, medullar, or bronchial aspirate. The immunological study was negative (further described in Table 1). Virologic tests for human immunodeficiency virus (HIV) and hepatitis virus were also negative. Imaging studies, such as abdominopelvic computed tomography (CT) scan, transthoracic echocardiogram, cervical echography, and laryngoscopy, reported no anomalies, as did the upper and lower gastrointestinal endoscopies. A liver biopsy yielded a discrete siderosis.

**Table 1:** Immunological studies RNP: ribonucleoprotein; SM: Smith; ANA: antinuclear antibodies; SSA/SSB: Sjögren's-syndrome-related antigen A/Sjögren's-syndrome-related antigen B; DNA: deoxyribonucleic acid; LKM: liver-kidney microsome

Anti-RNP	NEG.
Anti-SM	NEG.
Anti-mitochondrial	NEG.
Anti-smooth muscle	NEG.
Anti-LKM	NEG.
Anti-cardiolipin M/G	NEG.
Anti-B2 glycoprotein M/G	NEG.
ANA	NEG.
Anti-DNA ds	NEG.
Anti-SSA/SSB	NEG.
Rheumatoid factor	NEG.
Anti-streptolysin	NEG.

Once infectious, malignant and auto-immune causes for the syndrome were excluded and a diagnosis of AOSD was established. The patient fulfilled Yamaguchi’s criteria with three major criteria (the patient’s temperature didn’t reach 39ºC most of the time and was thus not used as a criterion) and three minor criteria. Prednisolone (1 mg/kg) was started, symptoms immediately improved, and the patient was discharged three days later with no symptoms. One month later, he remained asymptomatic and a tapering schedule was started and concluded six weeks later. One year after this episode, he remains asymptomatic, without any therapy, and having reported no similar events.

## Discussion

Most sets of diagnostic criteria (Goldman, Calabro, Cush, Reginato, Kahn, Yamaguchi, and Fautrel) classify AOSD as a diagnosis of exclusion [9].

Elevated serum ferritin is frequent in AOSD patients, being present in up to 70% of them, and these elevations correlate well with disease activity. Ferritin value upwards of five times the normal upper limit in the context of fever of unknown origin is highly suggestive of AOSD with a sensitivity of up to 80%, which can be further enhanced to 93% if combined with a proportion of glycosylated ferritin of less than 20%. This is, in fact, the only serological marker included in all criteria sets (appearing as a major criterion in Fautrel’s set). However, the limited availability of glycosylated ferritin limits its use as a useful serological marker.

Our patient was diagnosed according to Yamaguchi’s criteria with three major and three minor criteria (Table 2), but the diagnosis could also have been made using Fautrel’s criteria, even with the impossibility of using the glycosylated ferritin marker as a criterion (Table 3), or even using the more recent Crispin scale (Table 4) in which our patient would have scored 40 points (cut-off >30).

**Table 2:** Yamaguchi's criteria ANA: antinuclear antibodies; RF: rheumatoid factor; IgM: immunoglobulin M

Yamaguchi's criteria » Five or more criteria are required, of whom two or more must be major	
Major criteria	Fever >39 °C, lasting 1 week or longer
	Arthralgia or arthritis, lasting 2 weeks or longer
	Typical rash
	Leucocytosis >10.000/mm3 with >80% PMN cells
Minor criteria	Sore throat
	Recent development of significant lymphadenopathy
	Hepato/splenomegaly
	Abnormal liver function tests
	Negative ANA and RF (IgM)
Exclusion criteria	Infections
	Malignancies (mainly malignant lymphoma)
	Other rheumatic disease (mainly systemic vasculitis)

**Table 3:** Fautrel’s criteria PMN: polymorphonuclear

Fautrel’s criteria » Four or more major criteria are required, or three major and two minor criteria	
Major criteria	Spiking fever ≥39 °C
	Arthralgia
	Transient erythema
	Pharyngitis
	PMN cells ≥80%
	Glycosylated ferritin ≤20%
Minor criteria	Maculopapular rash
	Leucocytosis ≥10.000/mm^3^

**Table 4:** Crispin’s clinical scale PMN: polymorphonuclear; FOU: fever of unknown origin

Crispin’s clinical scale		
Findings	Description	Score
Arthritis	Presence of synovitis	10
Pharyngitis	Present during the beginning of the disease.	7
Still rash	Macular or maculopapular, pink-salmon, non-pruriginous rash that accompanies the fever.	5
Splenomegaly	Detected clinically or by imaging studies (>11 cm)	5
Neutrophilia	PMN cells ≥9.500/mm^3^	18
Total		45
≥ 30 points in patients with FOU allows for the diagnosis of AOSD without further studies (specificity ~98%)		

The apparent advantage of Crispin’s scale is that, in the setting of fever of unknown origin and by relying on clinical findings and routine workup, it can be calculated as early as admission, thus allowing for earlier diagnosis and establishing a positive diagnosis of AOSD without the need of further workup [9].

The absence of specific symptoms, which may not coincide in time, is one of the main obstacles in establishing a prompt diagnosis. In fact, most patients can be misdiagnosed and wrongly treated for a long period of time as showed in Carreno’s review of 20 cases, of which 80% of patients had a prior different diagnosis [10].

The recommended initial treatment choices in AOSD include non-steroid anti-inflammatory drugs (NSAIDs) or aspirin, but as the reported response rates are poor, they are now only used in mild cases of AOSD. Systemic corticoids are suggested as initial therapy but seem to be able to control symptoms in only about two-thirds of patients. Disease-modifying anti-rheumatic drugs (DMARDs) may be considered either as adjunctive initial therapy or as steroid-sparing agents and for preventing articular destruction. As sulfasalazine appears to have serious adverse reactions (ranging from gastrointestinal symptoms to severe myelosuppression), it should be avoided [11]. Methotrexate, cyclosporin, or hydroxychloroquine have been used in published case reports with apparent good outcomes and low adverse effects rates [12].

In resistant cases, biologic agents should be considered. Since the identification of immune pathological pathways involving mainly interleukin-1 (IL-1) beta and IL-18, but also IL-6 and TNF-alpha, agents targeting these cytokines (as anakinra - IL-1 or anti-tumor necrosis factor-alpha (anti-TNF-alpha agents)) have shown to be an effective treatment in AOSD in an isolated case report and small cohorts [13].

Fortunately, in this case, the patient presented with a monocyclic pattern with complete and sustained response to systemic corticoid therapy.

## Conclusions

Since AOSD is still an exclusion diagnosis, specific and positive diagnostics remain the main limitation to prompt diagnosis and the initiation of targeted therapies. AOSD is an extremely rare disease, and this was a particularly challenging case, in which every diagnostic option was progressively excluded. Even though promising, Crispin's clinical score needs further validation. The authors intend to increase awareness of this diagnostic hypothesis when approaching an adult with fever and arthralgia.
